# A Remotely Delivered, Peer-Led Physical Activity Intervention for Younger Breast Cancer Survivors (Pink Body Spirit): Protocol for a Feasibility Study and Mixed Methods Process Evaluation

**DOI:** 10.2196/18420

**Published:** 2020-07-08

**Authors:** Lauren S Weiner, Stori Nagel, H Irene Su, Samantha Hurst, Sheri J Hartman

**Affiliations:** 1 Department of Family Medicine & Public Health University of California, San Diego La Jolla, CA United States; 2 Moores Cancer Center University of California, San Diego La Jolla, CA United States; 3 Haus of Volta Murrieta, CA United States; 4 Department of Obstetrics, Gynecology, and Reproductive Sciences University of California, San Diego La Jolla, CA United States

**Keywords:** physical activity, cancer survivors, peer mentors, quality of life, pilot study, breast cancer, fitness trackers, mobile phone

## Abstract

**Background:**

Younger breast cancer survivors consistently report a greater impact of their cancer experience on quality of life compared with older survivors, including higher rates of body image disturbances, sexual dysfunction, and fatigue. One potential strategy to improve quality of life is through physical activity, but this has been understudied in younger breast cancer survivors, who often decrease their activity during and after cancer treatment.

**Objective:**

The aim of this study is to explore the feasibility and acceptability of a technology-based, remotely delivered, peer-led physical activity intervention for younger breast cancer survivors. We will also assess the preliminary impact of the intervention on changes in physical activity and multiple aspects of quality of life.

**Methods:**

This study is a community-academic partnership between University of California, San Diego and Haus of Volta, a nonprofit organization that promotes positive self-image in younger breast cancer survivors. This ongoing pilot study aims to recruit 30 younger breast cancer survivors across the United States (<55 years old, >6 months post primary cancer treatment, self-report <60 min of moderate-to-vigorous-intensity physical activity [MVPA]) into a 3-month peer-delivered, fully remote exercise program. Participants will complete 6 biweekly video chat sessions with a trained peer mentor, a fellow younger breast cancer survivor. Participants will receive a Fitbit Charge 3; weekly feedback on Fitbit data from their peer mentor; and access to a private, in-app Fitbit Community to provide and receive support from other participants and all peer mentors. At baseline, 3 months, and 6 months, participants will complete quality of life questionnaires, and MVPA will be measured using the ActiGraph accelerometer. Feasibility and acceptability will be explored through a mixed methods approach (ie, quantitative questionnaires and qualitative interviews). Intervention delivery and adaptations by peer mentors will be tracked through peer mentor self-evaluations and reflections, review of video-recorded mentoring sessions, and monthly templated reflections by the research team.

**Results:**

Recruitment began in September 2019. As of February 2020, the physical activity intervention is ongoing. Final measures are expected to occur in summer 2020.

**Conclusions:**

This study explores the potential for physical activity to improve sexual function, body image, and fatigue, key quality of life issues in younger breast cancer survivors. Using peer mentors extends our reach into the young survivor community. The detailed process evaluation of intervention delivery and adaptations by mentors could inform a future hybrid-effectiveness implementation trial. Finally, remote delivery with commercially available technology could promote broader dissemination.

**Trial Registration:**

ClinicalTrials.gov NCT04064892; https://clinicaltrials.gov/ct2/show/NCT04064892

**International Registered Report Identifier (IRRID):**

DERR1-10.2196/18420

## Introduction

### Background

Although women diagnosed with breast cancer at less than 50 years of age comprise fewer than 10% of all breast cancer survivors [1], they have lower survival rates than older survivors and experience unique health and psychosocial issues [2]. Younger age at diagnosis is a risk factor for more advanced disease, and younger women often undergo more aggressive treatment regimens [3]. Younger cancer survivors consistently report a higher impact of their cancer experience on quality of life compared with older survivors [4]. This is likely due to a combination of more aggressive treatments and being diagnosed at a time when they are in the midst of forming relationships, starting and raising families and/or caring for aging parents, and establishing a career and work-life balance [5-9]. Cancer-related physical and psychosocial changes can impact productivity at home and work, even after treatment concludes [10-12]. Key psychosocial concerns in this population include body image disturbances because of physical changes (eg, hair loss, weight gain, and surgical scars), sexual functioning, and fatigue [4,5,13-17]. To date, few evidence-based interventions have been identified for younger breast cancer survivors to improve these aspects of quality of life.

In older breast cancer survivors, physical activity reduces the risk of cancer recurrence and mortality [18-21]. Physical activity has also been shown to decrease fatigue and may ameliorate some of the most troubling problems of younger survivors, such as poor body image and sexual dysfunction [22-24]. Younger breast cancer survivors often reduce their activity levels during and after treatment [25] and are less likely to be active than similar-aged women without cancer [26]. Few physical activity interventions have been tested in younger cancer survivors [27-29], although younger survivors would be best served by interventions designed or adapted to target their specific needs. General barriers to exercise such as scheduling and lack of time may be particularly relevant for younger cancer survivors due to a combination of persistent side effects from aggressive treatment regimens and the many competing demands of this life stage [8]. In addition, given young survivors’ higher rates of depressive symptoms and fatigue, they may be more likely than older survivors to experience psychological barriers to activity (eg, low motivation and emotional distress) [8]. Taken together, these factors make it difficult for younger cancer survivors to start and maintain an exercise routine.

Intervention modalities that do not require in-person attendance and can be accessed anytime and anywhere, such as remotely delivered, technology-based physical activity interventions using wearable trackers and mobile phones, may help younger breast cancer survivors overcome barriers to exercising. Commercially available wearable activity trackers and their companion mobile apps (such as the Fitbit and Fitbit app) can facilitate numerous theory-based techniques shown to be associated with increasing exercise in cancer survivors, including self-monitoring, goal setting, and performance feedback on several activity-related metrics, including the number of steps taken, minutes of moderate-to-vigorous-intensity physical activity (MVPA), and continuous heart rate tracking [30-38]. Several studies using wearable trackers have demonstrated their efficacy, particularly when combined with traditional counseling, for increasing physical activity in the general population [39] and in older breast cancer survivors [40,41]. However, these trackers have not been tested with younger cancer survivors.

Adding peer support features to technology-based exercise interventions may enhance their effectiveness [42] and may be appealing to younger cancer survivors [7,43,44]. A mixed methods study of breast cancer survivors’ preferences for social support features within technology-supported, remotely delivered physical activity interventions revealed that survivors were highly interested in using technology-supported message boards to post questions, give and receive feedback, and share ideas with other survivors [42]. Similarly, survivors believed that sharing their exercise on a progress board or within a private community would help formalize goals, create a sense of accountability, and facilitate an understanding of their achievements relative to their peers [42]—behavioral theory–supported behavior change techniques [31]. Previous exercise intervention studies in young cancer survivors that have included online forums through study websites [27,29] or Facebook [28] have reported low engagement with the forums, perhaps due to lack of moderation [27] or moderation by a researcher [28,29]. Moderation of web- or app-based message boards by other younger cancer survivors could further enhance intervention effectiveness by increasing the likelihood that participants will engage with theory-based strategies designed to promote behavior change [42]. Further research is needed to determine feasible and acceptable strategies for increasing physical activity, and potential benefits for quality of life, among the understudied population of younger breast cancer survivors.

### Community-Academic Partnership

This project is a community-academic partnership between University of California, San Diego (UC San Diego) and Haus of Volta, a nonprofit organization based in Murrieta, California, that works with younger breast cancer survivors to promote positive body image and positive outlook on life after cancer. Coauthor SN is the founder and Chief Executive Officer of Haus of Volta, and SN and SJH are coprincipal investigators (PIs) of this project. The overall goal of this community-academic partnership is to develop an intervention that has the potential for dissemination and use by Haus of Volta. The project was developed using a community-based participatory research approach, which emphasizes the equal partnership and active involvement of community members and researchers in all aspects of the research process [45,46]. The initial research questions were developed based on SN’s personal experience with breast cancer and her outreach in the young survivor community. The physical activity intervention being tested in this pilot study is based on a 3-month theory- and technology-based intervention that successfully increased MVPA in older breast cancer survivors, delivered by a research interventionist in an academic comprehensive cancer center setting [47]. SN served as a primary resource in the design of the pilot intervention. As part of the development of the grant proposal, 5 members of the Haus of Volta Community Advisory Board field-tested the pilot intervention components and worked with the UC San Diego research team to make modifications to meet the needs of young breast cancer survivors, thereby enhancing the cultural sensitivity and relevance of the intervention to this population. These modifications included 100% remote delivery and the addition of the Pink Body Spirit Fitbit Community and the intervention toolbox (see the *3-Month Physical Activity Intervention* section). SN and the Haus of Volta Community Advisory Board reviewed the quality of life questionnaires (see the *Measures* section) to confirm that the questions were worded in ways that would address the issues and concerns young breast cancer survivors face. Any modifications to methods over the course of the project will be documented and collectively decided upon by Haus of Volta and UC San Diego. Haus of Volta will contribute to the interpretation and dissemination of the mixed methods findings.

### Study Objectives

This study will conduct a single-arm, pre-post pilot trial of Pink Body Spirit, a 3-month remotely delivered, peer-led physical activity intervention, in 30 younger breast cancer survivors. This community-academic partnership project has 3 aims: (1) explore the feasibility and acceptability of the pilot study methods using a parallel mixed methods approach (postintervention quantitative surveys and qualitative interviews), (2) assess the preliminary impact of the intervention on physical activity and quality of life, and (3) use a multimethod approach to explore the process of intervention delivery and adaptations by peer mentors. The overall goal is to develop an intervention that can be disseminated and used by Haus of Volta. This protocol paper reports on the rationale, methodology, and outcome measures of this ongoing community-academic partnership project.

## Methods

### Study Design and Overview

**Figure 1:**
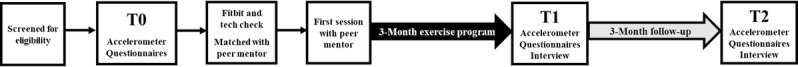
Flow diagram of the Pink Body Spirit pilot study.

Thirty younger breast cancer survivors (diagnosed when aged under 50 years and currently aged under 55 years, completed active treatment [ie, surgery, chemotherapy, and/or radiation], and self-reporting <60 min of MVPA per week) will be enrolled into a 3-month physical activity program, Pink Body Spirit. The Pink Body Spirit program is based on an intervention that was successful at increasing physical activity in older survivors delivered by a research interventionist in an academic comprehensive cancer center setting [40,47]. The Pink Body Spirit program will use peer mentors, motivational interviewing, and technology (Fitbit and Fitbit Community) to support behavior change. Five younger breast cancer survivors (diagnosed when aged under 50 years and currently aged under 55 years) have been trained as peer mentors to deliver the program to fellow younger survivors. To help address common barriers to exercise among young cancer survivors, the program is fully remote [8,48]. Participants will complete 6 video chat or phone sessions with their peer mentor biweekly and interact with their peer mentor and other participants through a private Fitbit Community at least weekly. To promote accountability, participants will be informed that their peer mentor will be able to see the physical activity data collected by the Fitbit. Between scheduled sessions, peer mentors will use real-time Fitbit data to identify participants in need of additional support to increase their exercise. At baseline (T0), 3 months (postintervention; T1), and 6 months (follow-up; T2), participants will complete quality of life questionnaires, and MVPA will be measured using the ActiGraph accelerometer. At T1 and T2, participants will also complete quantitative satisfaction questionnaires and qualitative interviews. Using a mixed methods approach (see *Mixed Methods Evaluation* section), findings from quantitative questionnaires and qualitative interviews will be triangulated to explore feasibility, acceptability, and satisfaction with the pilot study methods. Feasibility will be assessed through recruitment and retention metrics and adherence to intervention components (completion of intervention sessions, wearing the Fitbit, and posting in the Fitbit Community). A multimethod process evaluation will explore how the intervention was delivered and adaptations needed or made by peer mentors. Participants will be enrolled on a rolling basis, and intervention process data collected throughout the study will be used iteratively to provide feedback to peer mentors and adapt the program to meet the needs of the target population. The UC San Diego Institutional Review Board approved this study. Figure 1 shows the study flow diagram.

### Participants

#### Recruitment

Nationwide recruitment is being led by co-PI and younger breast cancer survivor SN. Recruitment will primarily occur through social media postings in groups tailored toward younger breast cancer survivors across the United States, including the Young Survival Coalition. Social media recruitment strategies have proven successful in recruiting young cancer survivors into research studies [49]. SN also gives presentations at many community events throughout Southern California and will use these community connections to support recruitment. Breast cancer oncologists and patient navigators at UC San Diego Moores Cancer Center have been asked to provide information to any patients who appear to meet the study criteria.

Eligible women must meet the following inclusion criteria: (1) breast cancer survivor diagnosed when aged between 18 and 49 years and currently aged between 18 and 54 years; (2) completed active treatment (specifically surgery, chemotherapy, and/or radiotherapy) at least six months before enrollment; (3) sedentary, defined as self-reporting less than 60 min of MVPA each week; (4) accessible by phone or video chat; and (5) have a Fitbit-compatible cellphone, tablet, or laptop with internet. Exclusion criteria include the following: (1) self-reported medical condition that could make it potentially unsafe to be in an unsupervised physical activity intervention as determined by the Physical Activity Readiness Questionnaire or self-reported peripheral neuropathy that interferes with ambulation, (2) currently pregnant, (3) inability to commit to a 3-month intervention schedule, or (4) prisoner.

#### Phone Screening

Women will be directed from the recruitment methods to call or email the study office to be phone screened for eligibility. Screener questions will include date of diagnosis, date when treatment was completed, self-reported activity, willingness to comply with study procedures, and access to and comfort with technology. The Physical Activity Readiness Questionnaire (PAR-Q) will be used to assess the risk of complications resulting from physical activity, including potential heart, joint, or bone problems [50]. Women who endorse any of the PAR-Q items will not be eligible for the trial. We will also ask about self-reported neuropathy and lymphedema that could make it difficult for a participant to exercise safely on their own. If eligibility is unclear after phone screening, the study physician (coauthor HIS) will review the data to determine if study enrollment is appropriate.

#### Informed Consent

Interested and eligible women will be provided additional information about the study and have any questions answered by the research staff. Potential participants will then be emailed a link to a web-based consent form through REDCap, a secure research database hosted on UC San Diego servers.

### Pink Body Spirit Peer Mentors

The Pink Body Spirit peer mentors are women who were diagnosed with breast cancer when they were less than 50 years old and have completed all active treatments for breast cancer (ie, surgery, chemotherapy, and/or radiation). The peer mentors were selected by co-PI SN and are being paid for their time by the grant funding this project. Peer mentors were trained by LSW and SJH in fall 2018 and spring 2019. All trainings were recorded for future use. Trainings included the following: Research Ethics, Privacy, and Data Safety; Exercising Safely and Adverse Events; Intervention Protocol and Delivery; and Motivational Interviewing (details provided below).

#### Research Ethics, Privacy, and Data Safety

All peer mentors completed the mandatory UC San Diego Collaborative Institutional Training Initiative (CITI) and Health Insurance Portability And Accountability (HIPAA) web-based trainings. An additional web-based Zoom training led by LSW applied key components covered in the CITI and HIPAA trainings to issues that are specifically relevant to this study.

#### Exercising Safely and Adverse Events

This web-based Zoom training led by LSW and SJH focused on exercising safely after breast cancer. Mock scenarios specifically relevant to this project were reviewed as a group to ensure that the mentors had gained the necessary knowledge of American College of Sports Medicine recommendations for cancer survivors [51] and how to help participants set safe and appropriate exercise goals. They were also trained on the study protocol to respond to and report adverse events. During biweekly mentoring sessions, peer mentors will ask participants if there have been any adverse events. Peer mentors were trained to encourage participants to seek medical attention and stop physical activity if necessary.

#### Intervention Protocol and Delivery

LSW worked with a peer mentor to create training videos of mock video chat mentoring sessions. The videos were distributed to all peer mentors along with a detailed script for the first mentoring session and the follow-up sessions. Peer mentors practiced the mentoring sessions with each other and individuals unfamiliar with the study. Before gaining approval to mentor study participants, peer mentors completed 2 mock sessions with LSW over Zoom video chat to demonstrate proficiency of the protocol content, logistics, and safety information. The approval process also included the verification of proper tracking of the mock sessions in the REDCap database.

#### Motivational Interviewing

Peer mentors were trained in motivational interviewing techniques for communicating with others, effective in supporting behavior change [52]. Motivational interviewing is a collaborative, goal-oriented method of communication with particular attention to the language of change. It is designed to strengthen an individual’s motivation for and movement toward a specific goal by eliciting and exploring the person’s own arguments for change. Peer mentors completed a standardized, publicly available, introductory course on motivational interviewing (10 hours [53]). The web-based training course, which could be completed anytime and anywhere, introduced peer mentors to the general principles of motivational interviewing (express empathy, develop discrepancy, roll with resistance, and support self-efficacy) and provided an overview of critical skills (open-ended questions, affirmations, reflective listening, and summaries). Following the web-based introductory training, peer mentors completed a study-specific in-person course on motivational interviewing led by co-PI and clinical psychologist SJH (4 hours). The in-person training focused on using motivational interviewing skills specifically to increase physical activity within the study protocol. During this training, peer mentors practiced mock visits with each other, and SJH and LSW provided real-time feedback on their use of motivational interviewing skills. Of note, the study team and 3 peer mentors based in Southern California elected to conduct this training in person, but 2 peer mentors were trained entirely remotely. The in-person training was recorded, and peer mentors who were trained remotely practiced mock visits with each other and LSW over Zoom video chat in preparation for final approval by LSW.

### 3-Month Physical Activity Intervention (Pink Body Spirit)

#### Technology Support Session and Peer Mentor Matching

The Pink Body Spirit intervention will begin after participants have completed the T0 measures. Participants will be mailed a Fitbit Charge 3 and will be matched with a peer mentor based on mutual schedule availability. Participants will then complete a brief technology support session via Zoom video conferencing with research staff. During this 15-min session, research staff will help participants set up their Fitbit and test the Zoom video conferencing in preparation for the peer mentoring sessions. Proactive technical support from research staff will allow peer mentors to focus on goal setting and behavior change strategies.

#### Peer Mentoring Sessions

**Figure 2:**
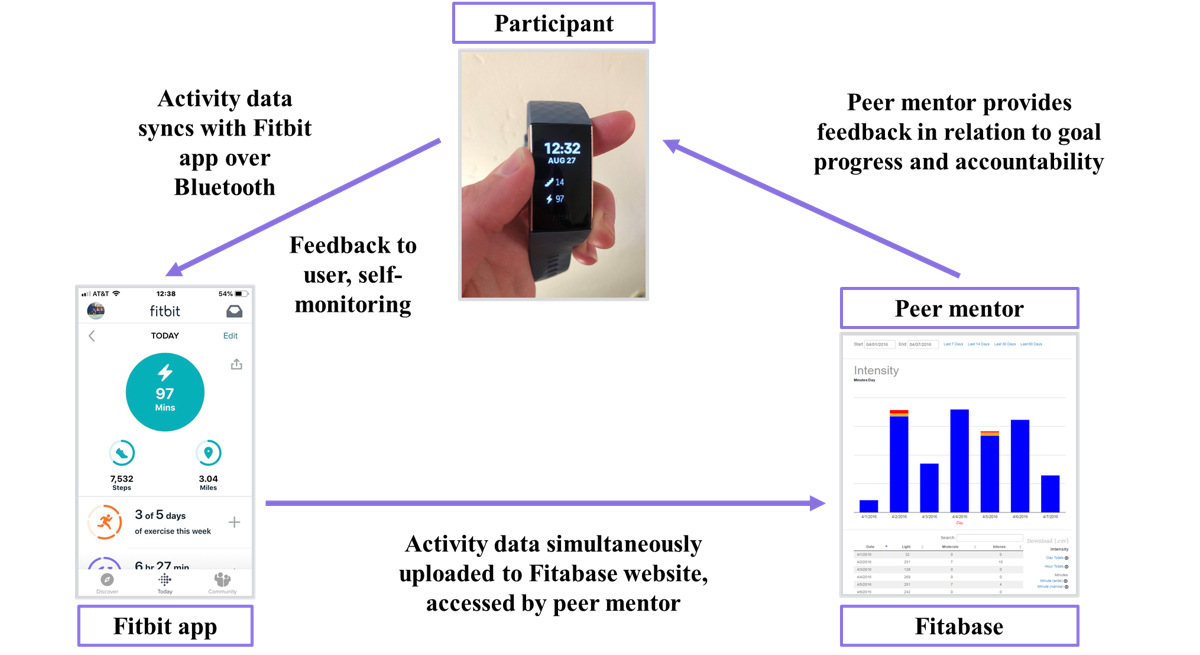
Visual depiction of how Fitbits will be used to support increasing physical activity in the Pink Body Spirit pilot study.

Participants will meet with their peer mentor 6 times over the course of the 3-month intervention. After completing the technology support session, participants will have an initial 45-min phone or video meeting (based on preference and capability) with their peer mentor. Initial meeting topics will include the following: (1) self-monitoring with the Fitbit; (2) in-app, private Fitbit Community; (3) goal setting; and (4) scheduling 5 biweekly follow-up sessions. The Pink Body Spirit intervention is guided by Control Theory [54] and Social Cognitive Theory [55]. Participants will learn how to self-monitor their physical activity using the Fitbit (see Fitbit details mentioned later; Figure 2). To promote self-efficacy, women will be encouraged to set specific, step-wise goals [39,47]. To increase self-efficacy and promote intention formation, peer mentors will use motivational interviewing techniques during the mentoring sessions. Participants will set a starting goal and a specific action plan to meet that goal. Goal setting will focus on safe and gradual increases in activity over time to meet the American College of Sports Medicine’s [51] and the American Cancer Society’s [56] guidelines for cancer survivors of engaging in at least 150 min of MVPA per week. To increase behavioral capability, peer mentors will teach participants how to use the Fitbit to monitor heart rate to determine moderate intensity and how to customize the display of their Fitbit app such that active minutes are displayed as the primary activity goal. A total of 5 follow-up video or phone sessions (approximately 20 min) will be scheduled biweekly over the 3-month program to check on progress, revise goals, and provide support. To promote accountability, participants will be informed that their peer mentor will be able to see the activity data collected by the Fitbit. Fitbit data will support performance feedback and goal review during biweekly follow-up sessions. To promote social support, improve rapport, and enhance continuity, participants will remain with their same peer mentor for the entire 3-month program.

Peer mentors will meet with LSW and SJH biweekly (1 hour) over Zoom video conferencing to gain support in delivering the intervention through discussion of how intervention sessions are going, problem solving for difficult participants, and practicing of skills. The use of the REDCap database, the Fitbit Community, safety, and adverse events will also be discussed. All intervention sessions will be video recorded. A random 50% of initial goal-setting sessions and a random 20% sample of follow-up sessions will be reviewed by the research staff. Review of the session recordings will serve a dual purpose: (1) to examine the use of motivational interviewing techniques and the safety of session content, which will be used to support peer mentors during biweekly supervision meetings, and (2) to examine adaptations from the study protocol. LSW will provide feedback to mentors based on the review of session recordings and information from participant qualitative interviews that are analyzed throughout the intervention using a rapid approach.

#### Fitbit and Associated Apps

Participants will receive a Fitbit Charge 3 (Fitbit, Inc.), an accelerometer-based activity monitor that provides real-time feedback on several activity-related metrics, including the number of steps taken, minutes of MVPA, and continuous heart rate tracking. The Fitbit was selected owing to its relatively low cost and ubiquity in the consumer wearables marketplace and in research studies. It is water resistant up to 50 m, has a battery life of approximately 1 week, and has 15 exercise modes to set goals and track statistics for activities as varied as swimming, yoga, and strength training. The Fitbit wirelessly uploads to the Fitbit website and smartphone app (Android, Apple, or Windows) that provide graphical visualizations of daily activity patterns. Participants will be instructed to wear their Fitbit daily and sync the tracker with the mobile app at least once per week so that their peer mentor can view their data in Fitabase (Small Steps Lab). Fitabase is a password-protected, web-based database program that collects physical activity and heart rate data from the Fitbit cloud. Through Fitabase, peer mentors can see graphs of daily light, moderate, and vigorous activity; date of last Fitbit sync; and Fitbit battery level for each of their mentees. Peer mentors will use the Fitbit data to support their scheduled follow-up sessions by identifying days with low activity where activity could be added and days with high activity to reinforce what is working well. Peer mentors will also be asked to check each of their participants’ Fitbit data through the Fitabase website at least weekly. Peer mentors will be trained to use Fitabase to identify general trends in activity and provide feedback to participants through email, text, or private Fitbit messages between biweekly mentoring sessions. Peer mentors will be provided with sample messages and encouraged to reach out to participants when (1) they decrease activity or do not meet their weekly goal, (2) they exceed their weekly goal, or (3) they have not worn or synced their Fitbit. This method of proactively reaching out to participants between scheduled sessions was highly successful and extremely liked in our previous study. Figure 2 illustrates how Fitbits will be used in the Pink Body Spirit intervention.

##### Fitbit Community

**Figure 3:**
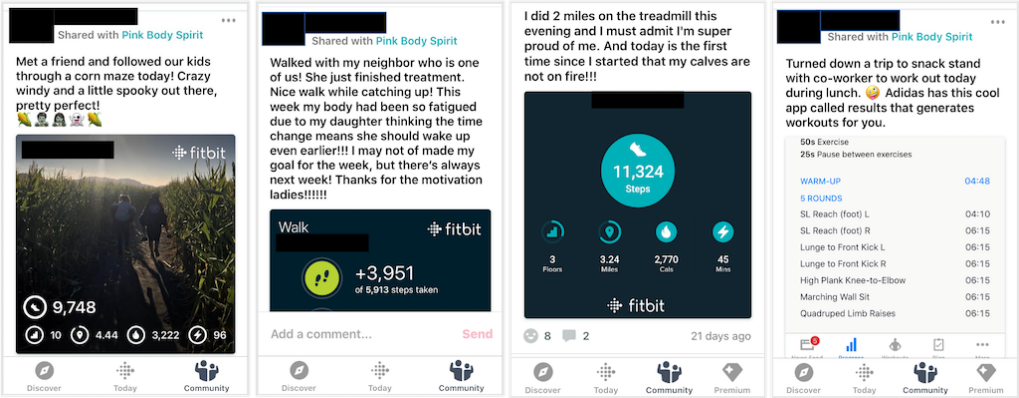
Sample posts from the private, in-app Pink Body Spirit Fitbit Community (note: app screenshots have been redacted to maintain confidentiality).

Participants will join a private Community group within the Fitbit mobile app where they can communicate with each other, see the activity Leaderboard (ie, activity levels of other participants), and gain support from all peer mentors. The Fitbit Community targets several theoretical constructs including social support; rewards and recognition (by other participants and mentors); and opportunities, barriers, and problem solving (through collective sharing of challenges, solutions, and resources). During the initial mentoring session, peer mentors will demonstrate how to access and post in the Fitbit Community. Step-by-step instructions for accessing and posting in the Fitbit Community will also be included with the Fitbit when mailed to participants. Participants will be asked to read and post in the in-app, private Fitbit Community at least once a week. To overcome the limitations of past studies in young cancer survivors that observed low engagement with online forums, the Fitbit Community will be moderated by peer mentors. Peer mentors will provide support to all participants through the Fitbit Community by checking it at least once a week and responding or posting as appropriate. Peer mentors will also reinforce participation in the Fitbit Community during biweekly mentoring sessions. Figure 3 shows example Fitbit Community posts.

#### Toolbox

Peer mentors will be encouraged to individualize the intervention through the use of a *toolbox* of exercise-related strategies and materials to help participants overcome barriers and achieve their unique exercise goals. Peer mentors will be able to spend up to US $40 on each participant to provide them with toolbox items such as fitness apps (free or paid), exercise materials for home-based workouts (eg, resistance bands, stability ball, or jump rope), and information about free workouts or exercise groups. Peer mentors can offer toolbox resources any time after the first session, and they must be linked with the participant’s individual exercise goal and action plan. Development of the toolbox was guided by the Haus of Volta Community Advisory Board’s field testing. The flexible, individualized toolbox approach has been a key component of numerous successful lifestyle interventions [57,58].

### Postintervention Follow-Up Period (Months 4 to 6)

The postintervention follow-up period in months 4 to 6 will focus on exploring the extent to which participants continue to engage with their mentors and different aspects of the Pink Body Spirit program beyond the initial 3-month intervention. There will be no scheduled contacts or video chat sessions with peer mentors during the follow-up period, but participants will still be encouraged to continue wearing and syncing their Fitbit to track their activity and participate in the Pink Body Spirit Fitbit Community. Peer mentors will be advised that they are not expected to check activity data on Fitabase once a mentee has entered the follow-up period. Although there are no planned sessions, some contacts may still occur. If a peer mentor chooses to communicate with a mentee during the follow-up period, it will be tracked in the REDCap database (date of support, mode [eg, email, Fitbit message, text, call, or Zoom video chat], and general reason for support).

### Measurement

#### Baseline Assessment (T0)

Participants will be emailed a link to a battery of web-based questionnaires consisting of quality of life measures, theoretical constructs for physical activity change, and self-reported muscle strengthening, stretching, and flexibility exercises. Participants will self-report demographics, general medical history, menstrual and reproductive history, and cancer history. Self-reported general medical history will include all current conditions and medications prescribed by a health care provider and dietary and nutritional supplements. Self-reported menstrual and reproductive history will include any history of hysterectomy and/or oopherectomy, age at first menstrual period, last menstrual period, and/or reason(s) period has stopped. Self-reported cancer history will include date of diagnosis, stage at diagnosis, type of surgery, date of surgery, cancer treatments (radiation, chemotherapy, and/or hormonal therapy, history and current), infusions for human epidermal growth factor receptor 2-positive breast cancer (eg, Herceptin, history and current), and lymphedema (history and current).

Once the questionnaires have been completed, participants will be mailed an ActiGraph GT3X+ accelerometer, a device that objectively measures activity, and instructed to wear it on the hip for the next 7 days during waking hours (for at least 12 hours per day). Two compliance reminders will be placed over the 7-day wear period. Once the ActiGraph has been worn for 7 days for at least 12 hours per day, participants will return it to the study office using a prepaid envelope. Upon receipt, ActiGraph data will be screened for sufficient wear time. If participants have not worn the device for at least 10 hours over 5 days or 50 hours over 4 days, they will be mailed another device and asked to rewear it. Participants will receive US $20 for completing the T0 measures. All T0 measures will be completed, and sufficient wear time will be verified before being matched with a peer mentor to begin the exercise program.

#### Postintervention Assessment (T1)

Three months after completing the first intervention session with their peer mentor, participants will repeat the web-based questionnaires completed at T0 and complete a 3-month quantitative satisfaction questionnaire. They will again be mailed an ActiGraph and asked to wear it for 7 days. The same wear and mail-back instructions and compliance protocol described above will be used. After returning the ActiGraph, participants will complete a 3-month qualitative interview. Participants will receive US $20 for completing the ActiGraph and questionnaire and US $10 for the individual interview.

#### Follow-Up Assessment (T2)

Six months after completing the first intervention session with their peer mentor, participants will repeat the web-based questionnaires completed at T0 and complete a 6-month quantitative satisfaction questionnaire. They will again be mailed an ActiGraph and asked to wear it for 7 days. The same wear and mail-back instructions and compliance protocol described above will be used. After returning the ActiGraph, participants will complete a 6-month qualitative interview. Participants will receive US $20 for completing the ActiGraph and questionnaire and US $10 for the individual interview.

### Measures and Outcomes

#### Feasibility and Acceptability

##### Recruitment and Retention

We will report recruitment and retention using a Consolidated Standards of Reporting Trials diagram. We will track those who contact the study office via phone or email. Specifically, we will track the number of women who contact the study office, referral source for each (eg, social media, community event, UC San Diego patient navigator), number of women phone screened, number of eligible participants, and number of participants enrolled in the study. We will compare the yield from different recruitment approaches and assess if any eligibility criteria have excluded a disproportionate number of otherwise eligible women.

##### Adherence

Adherence to the intervention will utilize several metrics: completion of intervention sessions, wearing the Fitbit, and posting in the Fitbit Community. Adherent will be defined as completing 2 out of 3 of the following activities: completing >75% of the sessions, wearing the Fitbit on >75% of the days, or posting in the Fitbit Community at least once per week for >75% of the weeks.

#### Mixed Methods Evaluation

A parallel mixed methods approach will be used to explore the feasibility, acceptability, and satisfaction of the Pink Body Spirit intervention from the participants’ perspectives. Quantitative satisfaction questionnaires will be used to gain breadth of understanding, and qualitative interviews will add depth of understanding [59].

##### Quantitative Satisfaction Questionnaire (Postintervention and Follow-Up)

Questionnaires will build off questions used to evaluate the previous intervention in older breast cancer survivors upon which the Pink Body Spirit program is based [41]. Topics will include overall ratings of the program, the extent to which the program provided motivation to start and continue exercising, and the likelihood of recommending the program to other younger survivors. A series of items using Likert-scale response options will assess self-reported frequency of use, satisfaction with, and helpfulness of each intervention component. Participants will be asked to rate different features of the Fitbit tracker, the Fitbit app, and the Fitbit Community. There will be a *did not use* option for each feature assessed. Participants will also be asked to rate their satisfaction with the number, length, and content of peer mentoring sessions as well as the Zoom video conferencing format. Open-ended questions will ask participants to describe their favorite and least favorite aspects of the program and provide suggestions for how to improve Pink Body Spirit. Additional topics for the T2 questionnaire will include continued use of intervention components, including the Fitbit, Fitbit Community, and toolbox items, after the 3-month intervention has formally concluded; the extent to which participants continue to communicate with peer mentors; and participants’ interest in being trained to serve as a peer mentor in a future study or program. To assess general attitudes toward Pink Body Spirit, we will use items from 2 validated, Likert-type measures of feasibility and acceptability [60]. Questionnaires will be piloted internally with the research and peer mentor teams before use.

##### Qualitative Interviews

At T1 and T2, individual, semistructured interviews will be conducted via Zoom video conferencing. Each interview will last approximately 45 min. Interviews will be video recorded and uploaded to the UC San Diego server. Interviews will explore topics such as overall perceptions of the intervention and specific components (eg, peer mentors and Fitbit), barriers and facilitators to behavior change, perceived health benefits (particularly in the targeted domains of body image, sexual functioning, and fatigue), the remote delivery format, and ways to improve the Pink Body Spirit program for other young breast cancer survivors. Semistructured interviews are proposed to keep the interview prioritized and targeted, but also allow for the flexibility to use additional probes to clarify responses and add depth to participants’ answers. Interview questions will be asked in a flexible order, and wording may be adapted to maximize each participant’s understanding. The interview guide will be piloted internally with the research team and with younger breast cancer survivors not involved in the study before administration. For consistency, LSW will conduct all interviews with the participants.

#### Physical Activity

##### ActiGraph GT3X+

The ActiGraph GT3X+ (ActiGraph, LLC) will be used to measure changes in minutes of MVPA from T0 to T1 and T1 to T2. This is the gold standard measure of free-living physical activity that provides blinded data collection. For 7 days around each measurement time point, participants will wear the ActiGraph GT3X+, a research grade, hip-worn accelerometer that measures movement and intensity of activity and that has been validated against heart rate telemetry and total energy expenditure. Physical activity data are not shown to the wearer. The GT3X+ provides second-by-second estimates of activity that can be categorized into minutes spent in sedentary, light, moderate, and vigorous activity using calibration thresholds. Sufficient wear time will be defined as 5 days with ≥600 min of wear time or 3000 min (50 hours) across 4 days. Time spent in sedentary behavior and light, moderate, and vigorous activity will be derived using Freedson cut-points [61].

##### Fitbit Charge 3

Data from the Fitbit Charge 3 will be used to calculate daily adherence to wearing the Fitbit throughout the intervention and to support peer mentoring. Participants will be asked to wear the Fitbit for as many hours per day as possible. Fitbit data will be wirelessly uploaded to the user’s personal Fitbit account and downloaded by our research team through Fitabase (Small Steps Labs), a web-based database program that collects physical activity and heart rate from the Fitbit cloud. Fitabase allows each participant’s Fitbit data to be batch downloaded at the 1-min level. Fitbit uses a proprietary algorithm to classify each minute as sedentary, light, moderate, or vigorous activity. Nonwear time will be determined based on the lack of heart rate or activity (steps or intensity) at any given minute [62]. Daily adherence to wearing the Fitbit will be defined as >5 min of wear [62]. A 5 min threshold for a valid Fitbit wear day will be used to avoid recording days where the Fitbit had simply been picked up and moved from one place to another, while simultaneously avoiding excluding days where the participant had purposefully worn the Fitbit. Fitbit data will not be used to measure changes in physical activity as participants cannot be blinded to the data collected.

#### Quality of Life

##### Body Image

Body image will be measured using the Body Image Scale (BIS), a 10-item scale developed for cancer survivors that measures perceptions of body disturbance related to cancer and its treatment [63]. Each item is rated on a 4-point Likert scale ranging from 1 (not at all) to 4 (very much). Higher scores indicate poorer body image. The BIS has shown high reliability (Cronbach α=.93), good clinical validity, and sensitivity to changes in psychosocial and physical activity interventions in older breast cancer survivors [63,64].

##### Sexual Function

Sexual function will be assessed using the Female Sexual Function Index (FSFI). The FSFI comprises 19 items assessing 6 domains of sexual functioning over the past 4 weeks: desire (2 items), arousal (4 items), lubrication (4 items), orgasm (3 items), satisfaction (3 items), and pain (3 items) [65]. The FSFI has been shown to be highly acceptable to breast cancer survivors and has good internal consistency (Cronbach α=.83-.96); test-retest reliability (*r*=0.74-0.86); and convergent, divergent, and discriminant validity [66]. The FSFI has been responsive to change in exercise intervention trials [67,68].

##### Fatigue

Fatigue will be assessed through the Patient-Reported Outcomes Measurement Information System Cancer Fatigue v1.0 measure [69]. The computer adaptive testing form will be used; participants will only complete 4-10 items out of the entire question bank. Higher scores indicate higher levels of fatigue. This instrument has shown responsiveness to change over time in prospective [69,70] and intervention [71] studies in cancer survivors.

### Process Evaluation of Peer Mentor Adaptations and Delivery

A multimethod approach will be used to explore the process of intervention delivery and adaptations made by peer mentors. These data will help us describe and understand barriers and facilitators to delivery, identify delivery strategies for use in a future study, and refine how to use delivery methods being tested in this study to maximize the potential for success in future studies. Data collection will occur during and after intervention delivery, and data from all sources will be used iteratively to improve intervention delivery by peer mentors. The data sources for this evaluation will include the following:

1. Peer mentor session feedback: After each mentoring session, peer mentors will electronically record their confidence and preparedness for delivering the session in REDCap. Questions were developed in partnership with a peer mentor and have been pilot tested and refined with the other peer mentors during mock training sessions. Questions will ask about the extent to which peer mentors used motivational interviewing skills during their session; the helpfulness of the motivational interviewing training in preparing them for the session; their confidence during the session; and general notes and reflections about the session, including any explicit adaptations made.
2. Zoom peer mentoring sessions will be recorded by peer mentors and uploaded to the UC San Diego server. As described above, a random 50% of initial goal-setting sessions and a random 20% sample of the follow-up sessions will be reviewed by LSW to support peer mentor supervision and safety and appropriateness of exercise goals set. A template will be used to guide the exploration of adaptations from the protocol, barriers and facilitators to intervention delivery, and other aspects of the intervention process.
3. LSW will write monthly reflections to document adaptations and process outcomes. Templated reflections will be based on discussions and interactions with multiple stakeholders (peer mentors, research team, and any informal feedback from participants). The reflections will help document key activities, events, and changes occurring over the course of the study.

### Statistical Analysis

#### Feasibility and Acceptability

We will explore how adherence varies between the 3 measures (completion of intervention sessions, wearing the Fitbit, and posting in the Fitbit Community) using descriptive statistics.

##### Postintervention and Follow-Up Quantitative Satisfaction Questionnaires

Descriptive statistics will be used to analyze the questionnaire responses. Responses will be reported as percentages or as mean (SD) as applicable. Open-ended responses will be coded using a standardized process. All open-ended questions and responses will be reviewed by 2 team members. Each response will be assigned at least one code, and codes will be nested into categories. Multiple coders and team consensus will be used to enhance the validity of the coding process and ensure that responses are accurately sorted into representative codes and categories.

##### Postintervention and Follow-Up Qualitative Interviews

Individual interviews will be video recorded and transcribed. A rapid assessment approach will be used to analyze qualitative interviews. The rapid assessment process is defined as “intensive, team-based, qualitative inquiry using triangulation, iterative data analysis, and additional data collection to quickly develop a *preliminary understanding* of a situation from the insider’s perspective” [72]. This method will allow us to feed data from participants back into the ongoing study to improve it. Data that could be fed back may include ways in which peer mentors could be more effective, tools that could be added to the toolbox, and barriers and facilitators that can be feasibly addressed to improve the intervention for other participants. In addition, the project timeline is relatively condensed, so a rapid approach, whereby qualitative analyses are conducted over the course of the intervention, may be more feasible than conducting all qualitative analyses after data collection has ended. Although traditional, in-depth qualitative analyses may be more constructivist, exploratory, and inductive, rapid analysis takes a more positivist approach that is explanatory and deductively oriented [73]. Coding will occur on a monthly basis. A team-based approach will be used to maximize the dependability and validity of the analyses and preliminary findings. As this is a minimally interpretive approach, individuals with limited qualitative training can participate [73]. The project will employ the five rapid analysis steps outlined by Dr Alison Hamilton [73].

##### Mixed Methods Interpretation

The quantitative surveys and qualitative interviews will be analyzed, and results will be reported separately. We will triangulate the quantitative and qualitative results to explore feasibility, acceptability, and satisfaction with the pilot study methods. The 2 sets of results (QUAN + QUAL) will be synthesized in the interpretation. The co-PI SN and Haus of Volta will contribute to interpretation.

#### Preliminary Intervention Impacts on Physical Activity and Quality of Life

Descriptive statistics (mean [SD] and n [%] where applicable) will be calculated for each variable of interest at T0, T1, and T2. Separate paired *t* tests will assess changes in each variable of interest from T0 to T1 and T1 to T2. The level of significance will be set at α=.05. From the means and SDs, Cohen *d* will be calculated to determine the effect size. These data will allow us to estimate the sample size needed to detect clinically significant intervention effects at an α of .05 and power of 0.80, should the program be tested in a larger efficacy trial.

#### Multimethod Process Evaluation of Intervention Delivery and Peer Mentor Adaptations

All findings will be triangulated to deepen our exploration of the intervention delivery process and adaptations made by peer mentors throughout the study. Peer mentor field notes will be downloaded from REDCap and reviewed by LSW. She will record memos throughout her review and will prepare a separate summary of the field notes for each of the 5 peer mentors. At the end of the study, the 5 summaries will be synthesized in a single narrative summary. LSW will follow up with peer mentors directly if any of the field notes require further clarification. Video recordings of sessions will be reviewed using a template checklist of predetermined variables. Data from each category of the template (eg, adaptations, barriers to intervention delivery, and facilitators to intervention delivery) will be transferred to a matrix. At the end of the study, LSW will develop a narrative summary for each category. Templated reflections will also be synthesized in a narrative summary at the end of the project.

## Results

Recruitment began in September 2019. As of February 2020, the physical activity intervention is ongoing. Final measures are expected to occur in summer 2020, with mixed methods data analysis and results available in winter 2021.

## Discussion

### Overview

This project leverages the complementary community-academic research expertise of Haus of Volta and UC San Diego to develop and deliver a novel intervention. Using a community-based participatory research approach, the UC San Diego research team worked closely with SN and Haus of Volta to adapt an evidence-based physical activity program to meet the needs of younger breast cancer survivors. This study will uniquely assess the potential for physical activity to improve body image and sexual function, key issues for younger breast cancer survivors that contribute to poor quality of life. Remote delivery using commercially available technology increases the potential for sustainability and dissemination.

### Limitations

Study limitations will be addressed whenever possible. One potential limitation is peer mentor drop out or turn over. Peer mentor selection, hiring, and caseloads will be determined by SN. If a peer mentor needs to discontinue mentoring before the end of the study, we will attempt to learn about their experience serving as a peer mentor. Valuable information will still be gained about the feasibility and acceptability of the program. Moreover, the small sample size may limit the generalizability of the findings, and the small group of younger women who choose to participate in this trial may not be representative of all younger breast cancer survivors. However, we will make the best use of our small study sample by using multiple qualitative and quantitative methods to take an in-depth look at the feasibility and acceptability of the Pink Body Spirit intervention and adaptations made by mentors in delivering the program. Exploring feasibility and acceptability using a mixed methods approach will help examine different facets of the intervention experience and provide a more complete picture of participants’ perspectives. In addition, using mixed methods will provide greater assurance about the validity of the research questions and the amount of data collected to answer these questions.

### Future Directions

There are several potential future directions after the completion of this pilot trial. If the intervention shows preliminary benefit for physical activity and/or quality of life, the investigators may choose to test the program in an adequately powered efficacy trial based on effect sizes from this pilot study. Alternatively, the detailed process evaluation of program delivery and adaptations made by the peer mentors could inform a future hybrid-effectiveness implementation trial. A hybrid trial would facilitate a formal evaluation of the implementation process. Another possible outcome is that Haus of Volta will bring the Pink Body Spirit program directly into the community. Through this project, we aim to increase the skills of younger breast cancer survivors so that they can better support each other in making healthy lifestyle choices and improving well-being, in line with Haus of Volta’s mission to promote positive self-image and health in younger breast cancer survivors. We elected to use a publicly available web-based training program for the majority of the motivational interviewing skills training and recorded the in-person training to support fully remote training of 2 peer mentors. The length of the training was not a barrier to engaging peer mentors and supported the development of introductory-level skills. Furthermore, all study-specific web-based Zoom trainings (eg, research ethics, exercising safely after breast cancer, and supporting safe exercise goal setting) were recorded for future use. The extensive peer mentor trainings have provided leadership skills to the peer mentors not only to deliver the intervention as part of this research study but also to continue serving as advisers to younger breast cancer survivors in various capacities after the study ends. Engaging Haus of Volta in interpretation of the results will enhance self-efficacy for conducting research and understanding and disseminating results. Part of the goal of reviewing and interpreting the results as a community-research partnership is to develop ways to empower and support the younger breast cancer survivor community to continue the physical activity program and peer mentoring in an ongoing manner. Haus of Volta could use pilot findings to apply for more funding to sustain the program. Pilot study results will be summarized in lay language for use by Haus of Volta and partnering organizations and for dissemination in survivor-centered settings, further increasing the impact and outreach in the younger breast cancer survivor community.

### Conclusions

The proposed study represents a novel contribution to the literature by providing rich information on ways to promote increasing physical activity and potential benefits to quality of life for younger breast cancer survivors. This project benefits from the combined knowledge of the community regarding the challenges faced by young breast cancer survivors and the research team’s expertise in conducting physical activity interventions and mixed methods evaluations. As smartphone ownership is reported by the majority of Americans and is increasing across all race/ethnicity, income, and age groups [74], this technology-supported, peer-delivered intervention has the potential for broad reach in geographically diverse, younger breast cancer survivors.

## Appendix

Multimedia Appendix 1 California Breast Cancer Research Program grant review.

## Abbreviations

BIS: Body Image Scale
CITI: Collaborative Institutional Training Initiative
FSFI: Female Sexual Function Index
HIPAA: Health Insurance Portability And Accountability
MVPA: moderate-to-vigorous-intensity physical activity
PAR-Q: Physical Activity Readiness Questionnaire
PI: principal investigator
T0: baseline
T1: postintervention
T2: follow-up
UC San Diego: University of California, San Diego
