# Supplementary material for: A Remotely Delivered, Peer-Led Physical Activity Intervention for Younger Breast Cancer Survivors (Pink Body Spirit): Protocol for a Feasibility Study and Mixed Methods Process Evaluation
Source: JMIR Res Protoc. 2020 Jul 8;9(7):e18420. doi: 10.2196/18420 (PMC7381067; doi:10.2196/18420)
Supplement: Multimedia Appendix 1 [file resprot_v9i7e18420_app1.pdf]

California Breast Cancer Research Program

Application Evaluation—CRC Pilot

Review Committee: Community Impact
Application #: 24AB-1300 & 1301
PI Names: Nagel & Hartman
Title: Physical activity intervention for young cancer survivors

---

SCIENTIFIC MERIT SCORES: (1 = BEST TO 9 = WORST)

Scientific merit component scores:

**Quality of the Research**	2.33
**Feasibility**	2.00
**Partnership**	1.92
**Community Benefit**	2.17

Average scientific merit score: 2.10
Committee rank within award type: 1st of 10 Pilot Proposals

Scoring Scale		
1 = Exceptional	4 = Very Good	7 = Fair
2 = Outstanding	5 = Good	8 = Marginal
3 = Excellent	6 = Satisfactory	9 = Poor

Part 1. Individual Reviewer Critiques:

Primary Reviewer

Summary of Aims and Methods (project overview)

The purpose of this study is to explore the benefits of physical activity for young breast cancer Survivors between the ages of 18-49. Young breast cancer survivors often have many negative effects from their cancer treatments at a time when they are just beginning their adult lives. Compared to older breast cancer survivors, they have a greater chance of cancer recurrence, sexual dysfunction, depression, fatigue, low self-esteem, and negative thoughts about their body.

The community-based co-PI partner of this study, Stori Nagel – founder of Haus of Volta, noticed that physical activity was helpful for her and many other young breast cancer survivors in readjusting to life after cancer treatments. Haus of Volta is interested in learning whether being physical active could help any of the common complaints among young survivors, and how to help fellow young breast cancer survivors be more active.

The academic-based co-PI partner of this study Sheri Hartman has implemented multiple technology-based physical activity interventions for breast cancer survivors and has worked with the community partner to collaboratively create an intervention that is specifically tailored to the needs and lifestyle of young breast cancer survivors in hopes to improve physical activity and quality of life for this population.

Using a mixed methods research design, the investigators hypothesize that:

1. a technology-based physical activity intervention delivered by trained peer mentors will be feasible and acceptable to breast cancer survivors between the ages of 18 and 49, and
2. the intervention will increase physical activity and improve multiple aspects of quality of life in young breast cancer survivors.

The study proposes a 3-month intervention trial to answer these questions. The following methods will be used:

First, the team will train 4 young breast cancer survivors to be peer mentors for the physical activity intervention.

Second, they will enroll 20 breast cancer survivors (under age of 50, greater than 6 months out of treatment, and currently not active) into a 12-week physical activity program.

The program includes a Fitbit and associated apps to encourage and track physical activity, and peer support through an online message board and regular phone/video calls. Each participant will receive tailored support from a trained peer mentor.

At the start of the intervention and twelve weeks later, participants will be asked to complete questionnaires measuring body image, self-esteem, depression, anxiety, fatigue, and sexual health. A small device (Actigraph) to measure physical activity will be mailed to participants to wear on their hip for 7 days at the start of the intervention and at twelve weeks. This device will be used to measure physical activity changes from pre- to post-intervention. The tool is a validated research-grade device that blinds participants to the knowledge of their activity levels as real time feedback is well-known to impact the engagement in physical activity.

Third, at the end of the intervention portion of the study, participants and peer mentors will be interviewed to get their perspective on the intervention and ways to improve it and make it sustainable.

This study represents the first trial from an ongoing collaboration to help young breast cancer survivors live healthier lives and improve many aspects of their quality of life. By using a remote-delivered intervention that incorporates peer support and technology, this program has potential for scalability and dissemination. Results from the proposed study will lead to a future trial where we hope to be able to test the efficacy of the PA intervention, in a large randomized trial, for increasing PA and improving quality of life. We will also use this pilot to learn how to empower the community of young breast cancer survivors and increase the capacity of Haus of Volta and the peer mentors to be able to support each other in making healthy behavior changes.

This program has significant potential to become sustainable, while increasing the skills of the community so that they can better support each other in making healthy lifestyle choices and improving well-being.

Primary reasons for recommended merit ratings (identify major strengths and weaknesses)

Strengths: The proposal is well-written, has a solid conceptual framework that is well-situated in the current literature.

The investigators have the skill sets and resources to do the study in the time frame and budget proposed.

Training will provide capacity building for the community partners.

If the intervention is deemed effective in improving not only physical activity parameters but the quality of life outcomes being measured, the benefit to the broader lay community could be vast. Technology based interventions are scalable and can be disseminated widely. Young women with breast cancer are left out of many research studies and this line of work addresses that gap.

Quality of the Research (strengths and weaknesses)

Strengths: The research questions are appropriate and important, particularly in light of the growing evidence that physical activity is associated with better cancer outcomes (e.g., recurrence and survival). Thus, it is timely to begin interventions that are acceptable to a population of younger breast cancer survivors, who are beset with worse quality of life, distress, fatigue, and depression than their peers who are older cancer survivors. Moreover, few physical activity interventions have focused on this group even though research shows that they are much less active than demographically similar non-cancer survivors.

The conceptual framework is clearly depicted and well-contextualized in the current literature.

The research design is well-thought out with appropriate measurements of outcome variables.

Power for Aim 2a is impressive with such a small sample.

Intervention is clearly described and plans are in place to modify as needed based on the qualitative feedback of participants and the peer mentors.

Weakness: Minor -- It would be useful to know slightly more about how the Actigraph data will be modeled. The application notes that time spent in sedentary, light, moderate, and vigorous PA will be derived with published cut points. Will a single aggregate score be used that is derived from the 7 days of baseline and post-intervention data?

Minor note – It would be useful to see a list of the interview questions to be posed in the qualitative phase to get a better sense of how acceptability of the intervention will be gauged.

Feasibility (strengths and weaknesses)

Strengths: Both partners have the capacity and skills and resources needed to carry out the proposed project.

The timeline provided is sound, and the procedures for the completion of the study are reasonable and should contribute to the likelihood of a successful outcome.

Weakness: Minor note -- Simulation studies show that the like hood of success with modeling random intercepts in data where there will so few subjects (a maximum of 20 people, 2 time points with potential missing data at time point 2) will potentially be problematic. There are often too many estimates to make with too few pieces of data, and convergence of the model may be a problem. A GEE model (i.e., marginal/population average model) is probably a better alternative in this case if convergence fails. This will still account for the clustering of repeated measurements (via a variety of correlation structure options), the analysis will retain all data available (even if both time points are not available); however, subject-specific random effects will not be modeled.

Partnership (strengths and weaknesses)

Strengths: Both partners appear equally invested in the success of the proposed project and have appropriate efforts and evidence of participation.

Aside from oversight of project implementation as academic co-PI, Dr. Hartman's skillsets are well-integrated into the project by providing training in motivational interviewing techniques to the four peer mentors. This training will be supplemented by a 10-hour online course in motivational interviewing. These new skills sets will add to capacity building among the community partner.

Weakness:

The project would not follow the PIs if either left their respective institutions. This would be a risk to the successful completion of the current project and the ability for a future project to be proposed from the results of this study.

Community Benefit (strengths and weaknesses)

Strengths: There is evidence of contributions from both partners integrated in the proposal and the plans for this continue throughout the study phase with the involvement of peer mentors and quality appraisal during the intervention implementation. Interviews with peer mentors and participants also show evidence of valuing the community partner's experience with the study. This enhances the likelihood of community benefit.

If the intervention is deemed effective in improving not only physical activity parameters, but also the quality of life outcomes being measured, the benefit to the broader lay community could be vast. Technology based interventions are scalable and widely disseminable. Young women with breast cancer are left out of many research studies and this line of work addresses that gap.

This project ideally lays the groundwork for the next stage of research and further collaborations between the community and academic partners to perform a fully powered randomized-controlled trial of the proposed intervention. Both partners are well-poised to see the current and future studies to fruition.

Weaknesses: Definition of the target community lacks complete clarity. That is, there is an inconsistency in an eligibility criterion. The methods portion of scientific abstract notes participants will be <50 years of age and **>6 years post treatment**. The methods portion of the lay abstract notes participants will be <50 years of age and **>6 months out of treatment**, while the program responsiveness section notes that participants will be <50 years of age and **<5 years post treatment**. The project narrative is in agreement with the lay abstract.

In short, clarification is needed regarding who would be eligible to participate, and justification for the decision should be provided.

The project fails to mention how it will target underrepresented women for participation in the project. Failure to do so, particularly with the use of an online intervention, may result in a majority white sample from middle income backgrounds. Marginalized folks from disadvantaged groups should be targeted in recruitment efforts.

One issue that comes to mind here is the need to ensure that all of your participants are safe to exercise. Physical activity is a life-enhancing health promoting behavior; however, it can also be abused or used in excess, especially in women who are struggling with body acceptance and disordered eating. The investigators might want to consider screening for problematic eating and exercise behaviors prior to enrollment. Thus, efforts should be made to enhance safety measures in the recruitment protocol. It is also important to be sure that the participants are not over exercising or using excessive exercise during the intervention. Peer mentors could be educated to be aware of the potential for this.

Budget, PI time commitment, project duration, and overlap (comments)

The budget is tight, as is typical with a small award (e.g., academic co-PI time commitment is on the low end of adequate). Nevertheless, the team has done a sufficient job of allocating resources in an appropriate and fair manner given the resource availability.

Project duration is appropriate.

Overlap issues: There seems to be some similarity between the proposed intervention and the academic co-PI's K-Award, however distinctions in outcomes (e.g., body image, sexual function) and certain intervention components (peer mentoring) have been made explicit.

The efforts noted on Dr. Hartman's CV are currently at 100%, with several in-kind projects ongoing as well. If the current project is awarded, she will have greater than 100% salary support for the first year of the study. If the PI is able to offer in-kind effort, funds might be better served in the community collaborators budget. Perhaps by increasing each peer mentor effort by 5 or even 10% (there is enough to do so).

Human and animal subjects risks (if any)

The uses of human participants in this study are appropriate to the project's aims. Risks are mostly low, and are adequately outlined, for the most part, in this section. However, as mentioned above, the investigators should consider how they will be ascertaining participants are safely using exercise. Prior to enrollment, screens for excessive exercise and eating disorders should be considered.

One drawback is that the project fails to mention how it will target underrepresented women for participation in the project. Failure to do so, particularly with the use of an online intervention, may result in a majority, or completely, white sample.

Secondary Reviewer

Summary of Aims and Methods (project overview)

This collaborative pilot study, led by Co-PIs Hartman from UCSD and Nagel from Haus of Volta, proposes to pilot test a 3-month pre-post study of a technology-based, remotely-delivered, peer-mentored physical activity intervention in increasing physical activity and improving quality of life (QOL) among young (18-49 years) breast cancer survivors. Investigators also propose to assess the feasibility and acceptability of the intervention in this group and strengthen the research partnership between UC San Diego and Haus of Volta.

The proposal addresses CBCRP's priority issue of "Community Impact of Breast Cancer". Its focus on younger survivors of breast cancer who are a health disparity group and the focus of the research on examining lifestyle behaviors and QOL make it responsive to this mechanism. Given the lack of peer to peer support based PA interventions in the young breast cancer survivor population, a feasibility pilot is appropriate.

Primary reasons for recommended merit ratings (identify major strengths and weaknesses)

This methodologically rigorous pilot intervention of physical activity in young breast cancer survivors is based on a significant premise of improving quality of life and reducing recurrence risk/morbidity/mortality from breast cancer in this health disparity population. The PIs' and coinvestigators' experience and expertise is complementary and increases confidence in the study's feasibility. The study need and idea of primary hypotheses originated with the community making it a high priority for the young breast cancer survivor community. This study will be one of the first to test a technology-based peer to peer mentoring intervention to increase PA and improve QOL in a young survivor population. If successful, findings from the current proposal can help design a larger randomized trial. Findings will also be broadly generalizable to young breast cancer survivors in California and the US. A minor weakness that detracts from the study is the lack of rationale or qualitative data for the peer to peer support part of the intervention which makes it more resource intensive.

Quality of the Research (strengths and weaknesses)

<u>Strengths:</u>

- Strong premise for conducting preliminary studies of technology-based PA interventions in young breast cancer survivors – an understudied group with different needs than older post-menopausal breast cancer survivors.
- Research gaps are well-articulated in the proposal and will provide preliminary data to support a larger clinical trial
- Community involvement in study hypothesis and study design is a strength.
- Intervention and outcomes based on a conceptual framework to improve QOL though physical activity
- Study design, eligibility, recruitment, intervention design and delivery including training of peer to peer mentors and Fitbit use are described in detail.
- Outcome measures are relevant to the young breast cancer survivors and measured through validated scales and objective accelerometer-based physical activity measurements.

<u>Weaknesses:</u>

- Minor: Rationale for peer to peer mentoring is not clearly articulated. Was it a component that the young survivor community requested? Was it based on prior qualitative data?

- Minor: Similarly, it is unclear whether prior qualitative data exists on this population to make motivational interviewing effective. What are the community perspectives on removing commonly identified barriers to PA in the young breast cancer survivor population? Is peer to peer mentoring the most acceptable or most efficient way to handle the barriers associated with PA?

Feasibility (strengths and weaknesses)

Strengths:

- Successful completion of the feasibility pilot will provide data to refine the intervention and design the larger RCT including providing sample size estimates
- Aim 3 will ensure that outcome and intervention components are culturally acceptable. Use of valid psychosocial instruments and objective PA measures is a strength.
- Feasibility and acceptability measurement methods are also described in detail
- Statistical analysis is appropriate and described in detail including age, stage of breast cancer and other covariates to be adjusted. Sample size and power justification is provided.

Weaknesses:

- None

Partnership (strengths and weaknesses)

Strengths:

- Involvement of the community partner and the young breast cancer community in framing the study question and in providing the peer to peer part of the intervention is a strength
- Plans for data ownership and sharing, handling disagreements, and plans for turnover of personnel are appropriate
- Involvement of the Community Advisory Board of Haus of Volta will ensure involvement from the broader community.
- Prior successful partnerships between UCSD investigators and PI Nagel is a strength.

Weaknesses:

- None

Community Benefit (strengths and weaknesses)

Strengths:

- Study aims directly responsive to needs of young breast cancer survivors – an understudied group
- Community and CAB involvement in peer to peer support training will increase community research capacity and engagement of the community in the current study
- Study is broadly generalizable to young breast cancer survivors in California and the US

Weaknesses:

- None

Budget, PI time commitment, project duration, and overlap (comments)

Budget, PI time commitment and project duration recommended as requested. No overlap identified.

Human and animal subjects risks (if any)

None

Advocate Reviewer

Overall Evaluation (identify major strengths and weaknesses)

STRENGTHS: Despite evidence of the value of physical activity to the well-being of breast cancer patients and survivors, most women find it difficult to sustain physical activity even at the minimal guideline standards. This study is based on the hypothesis that a technology-based physical activity intervention (FIT-bit and associated apps) delivered by trained peer mentors will result in increased physical activity and improve quality of life in young breast cancer survivors. It is widely acknowledged that young breast cancer survivors concerns, such as body image, self-esteem, sexuality and sexual health, reproductivity and others, may impact them differently than their older counterparts, possibly lowering their quality of life more significantly. An intervention that motivates increased physical activity and potentially improve multiple aspects of quality of life can be a valuable tool in treatment and survivorship. Given current societal norms, there is good reason to believe technology-based interventions will be the most successful models for young adults.

The concept for this application originated with the community partner, Stori Nagel, founder Haus of Volta, who then recruited the scientific research partners, Drs. Sheri Hartman and Hui-Chun Irene Su, from University of California, San Diego, setting the stage for a growing collaborative partnership and ensuring community participation throughout.

WEAKNESSES: There can be vast differences in concerns and responses of an 18 year old and a 50 year old (who isn't exactly "just beginning…..adult life"). Even the Young Survival Coalition (referenced here often because of Ms. Nagel's involvement in YSC) mostly speaks to concerns of women under 40 years of age, which suggests it might be a more appropriate cohort for this study. "….young breast cancer survivors (i.e., those under the age of 50) are often under-represented in research or excluded from research" needs a citation." Its possible under-representation due to recruitment issues has been identified, but it's unlikely to be for exclusion reasons.

Quality of the Research (strengths and weaknesses)

STRENGTHS - Specific efforts targeting improved quality of life for young breast cancer survivors are important, since young survivors may experience concerns, such as body image, self-esteem, sexuality and sexual health, reproductivity and others, differently than their older counterparts, possibly lowering their quality of life more significantly. The choice of testing a technological intervention (Fitbit and associated apps) for this population is grounded in the heavy and growing norm and use of technology in young adult populations as well as society in general.

The specific aims are clearly defined: 1) Explore the feasibility and acceptability of a technology-based, remotely-delivered, peer-moderated physical activity intervention for young breast cancer survivors; 2) Assess the intervention's impact on change in physical activity; 2b) Measure the intervention's effect on body image, self-esteem, depression, anxiety, fatigue, and sexual health; 3) Strengthen the partnership between Haus of Volta and UC San Diego researchers by further developing community relationships, skills, and infrastructure to support future research studies. The proposed intervention was field tested with members of the Haus of Volta community advisory board (CAB). Their feedback guided the design of this proposed study. The research plan includes step by step detail from training of peer mentors to data analysis, dissemination and building a strong collaborative partnership.

WEAKNESSES - 18-49 years seems to be a wide range of age for a definition of "young survivors, who may just be beginning their adult life." There has been an extensive literature search and identification of studies and papers related to PA and cancer, but there is no mention of an on-going clinical trial at the Fred Hutchinson Cancer Research Center, Improving Physical Activity in Young Adult Cancer Survivors (18-39), which also uses a Fitbit intervention, or another at the University of North Carolina (not restricted to young adults) that uses Fitbit as standard measurement of activity and text messaging as motivation in the intervention arm.

Feasibility (strengths and weaknesses)

STRENGTHS – Stori Nagel has clearly grown in very recent years in experience and dedication to helping young breast cancer survivors feel comfortable in their bodies, which, as she says, may be different than societal ideals, especially after treatments such as mastectomy. She became a leader of the Young survival Coalition (YSC) in 2014 and, furthering her commitment, founded Haus of Volta in 2015 and, subsequently, its community advisory board (CAB). Dr. Hartman's work focuses on development and implementation of technology based PA interventions for cancer survivors and those at increased risk for cancer. Though this will be their first time as co-PI's on a research study, their paths and work have intersected since they met at an YSC Summit, and they are both experienced in community-based research. Given the shared work they have both provided in developing the proposal, it is highly likely that the project will be completed within the time frame and budget.

WEAKNESSES – No major weaknesses. However, if the age eligibility remains 18-49, it would be informative to have results stratified by age groups.

Partnership (strengths and weaknesses)

STRENGTHS – The partners have included all of the elements to build a strong and lasting partnership. They have drawn on each other's experience to support equal control and participation in the project. The proposed research questions were conceptualized by Haus of Volta (Stori Nagel) and its community advisory board (CAB). They will continue to do outreach for recruitment of young breast survivors to the study, monitor study progress and participate in dissemination of results. Dr. Hartman (and Dr. Su) will conduct qualitative assessments and explore survivorships concerns unique to the young breast cancer survivors.

WEAKNESSES – The collaborative agreement doesn't explain where documents not containing personal health information (PHI) will be stored. Will Haus of Volta be responsible for storing any files? If an institution is dissolved how can the information remain property of a non-entity? Also, the agreement would benefit form more detail regarding replacement of a PI. "Replacements will be found with the support and approval of the PI that is remaining on the project" seems inadequate..

Community Benefit (strengths and weaknesses)

STRENGTHS – The concept for this application originated with Haus of Volta, the community partner. Its leader, Stori Nagel and its 5-member CAB are involved at every step of development of this application. It will remain involved in recruitment of study participants, dissemination of findings to a wide range of stakeholders, strengthening the collaborative partnership and building groundwork to support an application for a full research award. Haus of Volta has become a powerful force in promoting the well-being of young breast cancer survivors.

WEAKNESSES – Because Haus of Volta helps women, mostly under the age of 40, but not exclusively, according to the website, it needs to be clearer about the target community for any research endeavor. It's probably an attempt not to exclude anyone in its constituency that is the reason eligibility for this study is extended to age 49, but it does conflict with the usual research and treatment definition of young breast cancer patient or survivor.

Part 2. Summary of the Review Committee Discussion:

The review committee's discussion was consistent with the content of the written critiques. Overall, the scientific review committee believed that this is an important topic, the questions are clearly defined, the proposal is well-written, has a solid conceptual framework, and the investigators have the skill sets and resources to complete the project. They believed that the proposal is methodologically rigorous, and that there will be increased capacity among community members after the project concludes. The committee thought that it is very important contribution toward the project that the research question is coming from the community health centers. The committee believed the research plan included step by step detail from training of peer mentors to data analysis, dissemination and building a strong collaborative partnership. The committee felt that the weaknesses were minor, including lack of rationale/qualitative data for the peer to peer support part of the intervention; a study missing from the literature review (a study at Fred Hutchinson Cancer Research Center).

Part 3. Summary of the Programmatic Review and Funding Decision:
The council thought that the project was responsive to the CRC award type and CBCRP's priority areas. They believed that the project had strong dissemination and translation potential, and had strong advocacy involvement.

The application received a programmatic score of 17.4 (max = 21) and ranked 5th of 10 CRC Pilot applications evaluated. The application received an average scientific score of 2.10 and ranked 1st of 10 Pilot proposals.

Funding Recommendation: The council recommended this project for funding based on the combination of scientific merit and programmatic reviews and scores.
